# Psychometric evaluation of the interpersonal needs questionnaire in the Indonesian language

**DOI:** 10.1371/journal.pone.0279272

**Published:** 2022-12-16

**Authors:** Veranita Pandia, Efi Fitriana, Irvan Afriandi, Fredrick Dermawan Purba, Febrianti Santiardi Danasasmita, Abdullah Ichsan, Kent Pradana

**Affiliations:** 1 Department of Psychiatry, Faculty of Medicine, Universitas Padjadjaran–dr, Hasan Sadikin General Hospital, Bandung, Indonesia; 2 Department of General and Experimental Psychology, Faculty of Psychology, Universitas Padjadjaran, Bandung, Indonesia; 3 Department of Public Health, Faculty of Medicine, Universitas Padjadjaran, Bandung, Indonesia; 4 Department of Developmental Psychology, Faculty of Psychology, Universitas Padjadjaran, Bandung, Indonesia; Chiang Mai University, THAILAND

## Abstract

Suicide remains one of the leading causes of death among youths and the commonly reported associated risk factor is interpersonal needs, which consists of perceived burdensomeness and thwarted belongingness. To date, there is no validated interpersonal needs questionnaire in Indonesia. Therefore, this research aims to validate and evaluate the psychometric properties of the Interpersonal Needs Questionnaire (INQ-15) among adolescents and young adults in the Indonesian language. Based on the results, the INQ-15 has high internal consistency and test-retest reliability. It was also found to have satisfactory content and concurrent validity, as well as support two factor model of factorial validity. This implies that The Indonesian version of the INQ-15 is a valid and reliable questionnaire to measure the interpersonal needs among adolescents and young adults, both in clinical and research applications.

## Introduction

Suicide is the fourth-highest cause of death among teenagers and young adults aged 15 to 29 years, with a global rate of 9.0 cases per 100,000 people. Globally, the suicide rate in males with 12.6 cases per 100,000 is higher than in females with 5.4 cases per 100,000 [1]. Approximately 77% of suicide-related deaths occur in low to middle-income nations, including Indonesia, with a prevalence of 88% among adolescents [1]. In 2016, about 2.9 suicide deaths per 100,000 population occurred in Indonesia, while 4.75% of adolescents had suicidal ideation [2, 3]. Furthermore, The Jed Foundation (JED) reported in 2020 that 84% of high school counselors found an increase in mental health concerns among their students over the past five years. This problem is often associated with high academic pressures and expectations for their futures. Students felt they did not know how to cope with stress, where to seek help for mental health issues, or even aware of suicidal ideation signs [4]. As for college students, challenges vary from academic demands, adaptation to new environments, adjustment of learning styles, and time management skills. In some ways, the student population is more vulnerable than other groups of young people. Students with mental problems potentially have some considerable risk of academic failure. Moreover, mental illnesses in adolescents and young adults can sometimes be challenging to diagnose in their early stages [5].

There are also limited strategies to reduce this high suicide prevalence and improve the unmet needs in suicidal behavior management. Current suicidal strategy only focuses on the individual rather than the group or institutional level [6]. Since the suicidal behavior progresses from suicidal ideation and plans about taking one’s life through suicidal acts with increasing lethality and intent [7], one of the most effective ways to prevent this behavior is by early detection using simple screening tools. A meta-analysis research conducted in 2018 showed that psychoeducation and screening conducted in schools are effective to reduce the frequency of suicidal ideation in adolescents and young adults [8]. Therefore, screening for suicidal ideation is a high concern in mental health and requires a great deal of focus [9].

According to interpersonal theory, suicidal ideation consists of passive and active ideation. It is said to be passive when the manifestation is still limited to cognition, while the active ideation is characterized by an active desire or behavior to end life. Passive suicidal ideation can develop into active when there is hopelessness in an individual. Similarly, active ideation can develop into suicidal behavior when there is an acquired capability for suicide. This implies that suicide prevention efforts must be performed before the emergence of passive suicidal ideation to inhibit its development into active, by preventing or managing hopelessness in an individual. People who have passive suicidal ideation are characterized by low feelings of belonging (low belongingness) and the feeling of being a burden (burdensomeness) [10].

Suicidal behavior process begins with ideation, planning, attempt, and finally death. In the Interpersonal Theory of Suicide, an attempt requires desire and capability [10]. Consequently, in suicide prevention, the identification of risk factors associated with the desire is essential. Insufficient interpersonal needs consisting of Thwarted Belongingness (TB) and Perceived Burdensomeness (PB) will contribute to the development of suicidal ideation [10–12]. TB is defined as a psychologically-painful mental state demonstrated when the fundamental need for connectedness is unmet. The dimension of TB consists of loneliness and reciprocal care. Furthermore, the component of this dimension includes self-reported loneliness, having fewer friends, living alone, incomplete family members, social withdrawal, and conflict within the family. Loneliness, insufficient social support, and social isolation are signs of unfulfilled interpersonal needs in the TB domain. Meanwhile, perceived burdensomeness (PB) is a mental state characterized by apperceptions that others will “be better off if I were gone” [10]. This condition potentially develops in some situations such as people with chronic illness, unemployed, or having a family conflict [11, 12]. Previously, it was found that lower PB and lower TB were associated with decreased depressive symptoms [13, 14]. Moreover, PB is a mediator between anxiety and psychological distress with suicidal ideation [15, 16]. Similar to PB, TB is also correlated with anxiety [17]. In more depth, both emotional regulation and PB, as well as TB, is observed as key mechanism underlying suicide risk [18]. One of the measurements that can be used to detect risk and protective factors for suicidal ideation includes The Interpersonal Needs Questionnaire (INQ) [19].

The INQ-15 was developed by Van Orden, et al to measure TB and PB [19]. Several adaptations and psychometric evaluations of INQ-15 has been conducted in adolescents and young adult population in various Asian countries, including Korea, China, and Singapore [11, 20–24]. The Singapore and Korean versions of INQ have good internal consistency (with Cronbach’s alpha coefficient of 0.93 and 0.9). The Singapore version INQ found good fit two factor model (χ2(88) = 373.24, p< .001, SRMR = .07, RMSEA = .10, CFI = .93, TLI = .92) and good convergent validity with The Depression Anxiety Stress Scale (DASS) [21]. Moreover, the Korean version has good convergent validity with Beck Depression Inventory-II (BDI-II) and good discriminant validity with the Reasons for Living Inventory (RFL). This study analyze 5 factor models. In the result, there were 3 models which shown good fit [21, 25]. INQ-15 measurement, is the recommended version that demonstrates good internal consistency and is validated psychometrically to be used in further research [22].

However, the adaptation into the Indonesian language has not been performed and no psychometric evaluation has been conducted to date. Psychometric evaluation was used to integrate the previous and existing theories regarding INQ and interpersonal theories of suicide, as well as to provide detailed information on the validity and reliability of the INQ screening tool. Therefore, this research aims to evaluate the psychometric properties of the INQ-15 in the Indonesian language, especially within the adolescent population and young adults to identify risk factors for suicidal ideation and develop further strategies to prevent suicide in the future.

## Method

### Design and participants

The consecutive sampling method was used, while the sample size was based on the formula of estimated suicide prevalence, according to a research in Indonesia which found that the prevalence of suicidal ideation in students was 18,3% [26]. The calculation was performed using a 95% confidence interval with a 5% margin of error. The minimum sample size estimation was 227 for both high school and college students. The sample size was based on the rule of thumb the ratio of observations to variables is to have at least five times as many observations as variables, and a ratio of 10:1 is considered a more suitable sample size. Even a minimum of 20 samples for each variable is suggested by some studies [27].

The participants were 343 or 47% students from 39 senior high schools and 395 or 53% from 14 universities in Bandung, West Java Province, Indonesia. The mean age of the senior high school students was 17.05 years with SD = 0.75; range: 15–19 years. Meanwhile, that of the university students was 20.28 years with SD = 1.07; range: 17–29 years, also, the majority of the participants were female namely 77%. According to previous reports, bias can be reduced by having a bigger sample size of participants [21]. The other consideration is to use comprehensive statistical methods, such as CFA to analyze the reliability and validity. From the procedure of data collection, using a digital form *Surveymonkey* filled during separate zoom sessions led by Psychiatrists and Psychologists in the research team for all participants could reduce the bias.

### Measures

#### Interpersonal Needs Questionnaire-15 (INQ-15)

The INQ-15 is a self-report questionnaire derived from The Interpersonal Theory of Suicide to measure thwarted belongingness and perceived burdensomeness. These two factors are the ultimate cause of suicidal ideation [19]. Hill, et al. found that the result of the confirmatory factor analysis of INQ-15 are consistent and are the best compared to other versions with Cronbach alpha scores ranging from 0.85 to 0.90 for PB and 0.81 to 0.87 for TB, indicating strong internal consistency [22]. A similar research among young adults in Singapore showed sensible internal consistency results, specifically Cronbach’s Alpha values of 0.95 for PB and 0.89 for TB [21]. The perceived burdensomeness subscale consists of 6 items, while the thwarted burdensomeness subscale consists of 9. Moreover, a Likert scale of 1 to 7 was used which consists of "not at all true for me" to "very true for me".

#### The Depression Anxiety Stress Scale 18 (DASS-18)

The Depression Anxiety Stress Scale (DASS) questionnaire measures three dimensions of negative emotions, namely depression (DASS-D), anxiety (DASS-A), and stress (DASS-S). It originally consists of 42 items that are negative emotional symptoms [28], subsequently, DASS-21 was developed and in 2013, Oei et al further formulated DASS-18 [29]. Based on research in the Indonesian sample, DASS-18 has good internal validity, with Cronbach’s alpha values for the depression scale of 0,87; anxiety 0,85, and stress 0,72. Therefore, it was considered to be more suitable for Asian populations, including Indonesia [29]. An example item of DASS-18 is “I felt I was not worth much as a person” and “I felt that life was meaningless”. A Likert scale of 0 to 3 was used which consists of "did not apply to me at all" to "very much" or "most of the time". A higher score indicates more severe emotional distress [28, 29], and the Cronbach Alpha coefficient was 0.93.

#### Beck Depression Inventory-II (BDI-II)

The Beck Depression Inventory-II (BDI-II) is a 21-item self-administered inventory to determine the severity of depressive symptoms [30]. University students were asked to rate each item using one of four response options based on the degree of the symptoms they had in the previous weeks, ranging from no to severe. Each answer option was rated from 0 (not) to 3 (severe). Suicidal thoughts or wishes are an example of BDI-II item, with responses ranging from "*I don’t have any thoughts of killing myself*" for option score 0 to "*I would kill myself if I had the chance*" for 3. Furthermore, the questionnaire consists of 3 subscales, which are cognitive, somatic, and effective [30]. Wang & Gorenstein in 2013 reportedly used the BDI-II in multiple languages with a mean Cronbach’s Alpha of 0.9, ranging from 0.83 to 0.96 [31]. The Indonesian version was validated by Ginting, et al. in 2013 [32], and the Cronbach Alpha coefficient obtained was 0.94.

#### Children’s Depression Inventory (CDI)

The Children’s Depression Inventory (CDI) is a 27-item questionnaire used to assess depression symptoms experienced in the previous two weeks [33]. The questionnaire was filled out by the high school students. For each symptom, participants were asked to select one of three alternative statements: "*I don’t think about committing suicide*," "*I think about committing suicide*," or "*I wish to commit suicide*." Each remark was given a value of 0, 1, or 2, with higher scores indicating more depression. This questionnaire consists of five subscales, which are negative mood, interpersonal difficulties, ineffectiveness, anhedonia, and negative self-esteem. In both Western and Asian populations, CDI has proven to be a reliable tool for diagnosing depressive symptoms [34–38]. Additionally, the Cronbach Alpha coefficient for CDI obtained in this research was 0.87.

#### The Brief Reasons for Living Inventory for Adolescents (BRFL-A)

The Brief Reasons for Living Inventory for Adolescents (BRFL-A) is a 14-item questionnaire used to assess the reasons that a person will decide not to end their life despite feeling suicidal [39, 40]. It consists of five subscales, which are fear of social disapproval (FSD), moral objections (MO), survival and coping beliefs (SCB), responsibility to family (RF), and fear of suicide (FS). For each item, participants were asked to select one of five alternatives, ranging from “not at all important” for option score 1 to “extremely important” for 6. An example item of BRFL-A is “I believe I can find other solutions to my problems”. The total BRFL-A score had a Cronbach’s alpha of 0.75, while coefficients alpha for each subscale were 0.80 for FSD, 0.79 MO, 0.76 SCB, 0.74 RF, and 0.67 for FS [39]. Meanwhile, the Cronbach Alpha coefficient for BRFL-A was 0.77.

#### The Difficulties in Emotion Regulation Scale–Short Form (DERS-SF)

The Difficulties in Emotion Regulation Scale (DERS) is a tool that is frequently used to assess a person’s ability to regulate their emotions [41]. The questionnaire originally comprised 36 items. However, it has now been developed into shortened versions, namely Difficulties in Emotion Regulation Scale–Short Form (DERS-SF). It has a correlation coefficient of 0.90 to 0.97, indicating that DERS and DERS-SF share 81–84% of their variation. The total score had a Cronbach’s alpha coefficient of 0.70, with the six subscales ranging from 0.78 to 0.91. Strategies, non-acceptance, impulse, aims, awareness, and clarity are the six subscales of the DERS-SF. The values of three items were reversed, namely numbers 1, 4, and 6 which are awareness subscales. For each item, participants were asked to select one of five alternatives, ranging from “almost never” for option score 1 to “almost always” for 5. An example item of DERS-SF is”*When I’m upset*, *I feel guilty for feeling that way*”. Scales can be scored using sums or averages of items, all subscales were also scored, hence, higher values reflect greater difficulty with emotion regulation [42], and the Cronbach’s Alpha Coefficient obtained for DERS-SF was 0.89.

### Procedure

The subjects were recruited from senior high schools and universities in Bandung, Indonesia. The recruitment was performed through school teachers, and flyer ads, then those who were interested in participating were contacted. Students willing to participate signed a Letter of Approval in the digital Informed Consent form. The Informed Consent for high school students was signed by students and parents, while for university students, it was signed by the specific individual. The participants provided their informed consent and completed the INQ-15, DASS-21, BDI-II, and CDI questionnaires on an online survey using Surveymonkey which was supervised by Zoom video conference.

Approval was obtained to translate and validate the INQ-15 questionnaire from Kim Van Orden on 22 February 2020. It was translated into Indonesian and then backward-translated into English. Two expert translators translated the original version into the Indonesian language, and two more translators back-translated it into English, then the original was compared to the back-translated version. However, in this Indonesian version, some items load on a different component than the original version to fit into the context of Indonesian people. The validation process was similar to other measurement tools in Asian countries with unique cultural and demographic characteristics, such as Thailand [43, 44]. These differences can be explained by the characteristics of participants, particularly their cultural differences and demographics between the Eastern and Western contexts [45]. Furthermore, five experts from the departments of psychiatry, psychology, public health, and doctors discussed the findings as part of Guidelines for the Process of Cross-Cultural Adaptation Stage IV: Expert Committee review to reach consensus on discrepancies, also based on International Test Commission Guideline for Translating and Adapting Test [46, 47]. These experts is needed as part of Test Development Guidelines, to ensure the translation and adaptation processes consider linguistic, psychological, and cultural differences in targeted population [47]. The experts corrected any inconsistencies, while cognitive interview sessions with high school and university students were undertaken to create the final version. Ethical approval was obtained from the Universitas Padjadjaran Research Ethics Committee (No.1135/UN6.KEP/EC/2020).

### Data analysis

The mean and standard deviation were calculated to describe the data, these two statistics were used to determine the number of individuals who had significant suicidal ideation. Furthermore, the T-test was used to describe the relationship between INQ-15, gender, and education level. The step of data analysis performed included the psychometric aspects of reliability, as well as content validity, convergent validity, discriminant validity, and factorial validity or construct validity.

The Cronbach’s alpha test was also used to evaluate the internal reliability using guidelines from Cicchetti to interpret the Alpha’s score namely below 0.70 = unacceptable; 0.70–0.79 = fair; 0.80–0.89 = good; 0.90 and above = excellent [48]. Moreover, the test-retest reliability coefficient was calculated using Pearson product-moment correlation between the first and the second time measure. The interval between the test and retest was approximately one to two weeks after the first time of measurement, while a correlation of 0.1 to 0.3 is considered weak, 0.4 to 0.6 moderate, 0.7 to 0.9 strong, and 1.0 perfect [49].

The content validity index (CVI) was used to measure INQ-15 based on expert judgments. Convergent validity was evaluated using Pearson product-moment correlation with other measurement tools such as DASS-18, BDI-II for university students, CDI for senior high school students, and DERS-SF. Moreover, discriminant validity was evaluated using Pearson Product moment correlation with BRFL-A, while LISREL 10.3 was used to assess the confirmatory factor analysis.

The CFA model was assessed using multiple measures of goodness-of-fit such as χ^2^, the adjusted goodness-of-fit index (AGFI), comparative fit index (CFI), the non-normed fit index (NNFI), root mean squared error of approximation (RMSEA), and standardized root means square residual (SRMR). AGFI, CFI, and NNFI values ranged from 0 to 1.0, with values > 0.9 indicating a good fit to the data. Meanwhile, for RMSEA and SRMR, smaller values indicate a better fit, with values < 0.10 indicating a good fit and values < 0.05 a very good fit [50].

## Result

### Descriptive statistics of INQ-15

The interpersonal needs questionnaire’s score ranged from 15 to 99, with a mean of 44.56 and a standard deviation of 16.08. The dimension of perceived burdensomeness and thwarted belongingness had a mean of 14.61 and 29.85, respectively as shown in Table 1. There was a significant difference in the perceived burdensomeness between males and females, wherein the female respondents showed higher total average scores. There was also a significant difference between students from senior high school and university levels. The INQ-15 total score and the dimension of thwarted belongingness showed no significant difference neither for gender nor education level.

**Table 1:** Mean, standard deviation, and the t-test comparison.

INQ and dimension	Gender and education	M (SD)	t_(*df*)_	p-value
INQ total	Male	42.87 (14.20)	t_(313.63)_ = -1.69	0.09
	Female	45.06 (16.59)		
Perceived burdensomeness	Male	13.30 (6.89)	t_(322.43)_ = -2.66	0.01
	Female	14.99 (8.27)		
Thwarted belongingness	Male	29.57 (9.31)	t_(300.82)_ = -0.60	0.37
	Female	30.07 (10.40)		
INQ total	Senior high school student	43.98 (15.03)	t_(735.66)_ = -0.91	0.61
	University student	45.06 (16.94)		
Perceived burdensomeness	Senior high school student	13.46 (7.55)	t_(736)_ = -3.65	0.00
	University student	15.60 (8.25)		
Thwarted belongingness	Senior high school student	30.52 (9.74)	t_(736)_ = 1.41	0.16
	University student	29.46 (10.48)		

### Reliability

Based on the data from 738 participants, the INQ-15 had high internal consistency for all 15 items, as demonstrated by Cronbach’s alpha coefficient of 0.91. The correlation between each item score and the corrected total item ranged from 0.39 to 0.71. The Cronbach’s alpha coefficient for the dimension of PB was 0.92 for the 6 items, while that of TB was 0.85 for the 9 items. The corrected total items correlation for PB ranged between 0.70 to 0.82 and for TB 0.47 to 0.71. Furthermore, the test-retest reliability was analyzed based on the retest data collected approximately one to two weeks after the first time of measurement. Approximately 70% of the participants participated in the second measurement. The test-retest reliability coefficient was 0.83, which was categorized as strong.

### Content validity

The content validity of the INQ-15 was established after evaluation by an expert panel, revealing a high degree of relevance. This was indicated by the high item content validity index (I-CVI = 0.99) and high scale content validity index S-CVI = 0.93. In the focus group discussion, the participants remarked that the INQ-15 was easy to comprehend and had satisfactory face validity. In addition, the pilot test confirmed that it was acceptable in terms of administration time and easy to understand.

### Convergent validity

The convergent validity was established by elaborating the associations with several other questionnaires that are convergent with INQ-15, namely DASS-18, BDI-II, CDI, and DERS. DASS-18 was designed to measure emotional distress, BDI-II for the severity of depressive symptoms in young adults, and CDI was used to assess depression symptoms in adolescents. The DERS’s score strongly correlated with psychopathology and inversely with measures of psychological well-being.

As shown in Table 2, both INQ-15 total and its subscales scores had a significant association with those questionnaires, namely (1) DASS-18 total and subscales scores, with correlation coefficient ranging from 0.40–0.65. (2) BDI-II 0.47–0.69. (3) CDI 0.35–0.72, and (4) DERS 0.24–0.63. This result indicates that INQ-15 has good concurrent validity.

**Table 2:** The correlation coefficient between INQ-15 and the criterion variables.

		INQ		
		*Total*	*Perceived burdensomeness*	*Thwarted belongingness*
DASS	*Total*	0.65**	0.64**	0.53**
	*Stress*	0.52**	0.54**	0.40**
	*Anxiety*	0.55**	0.54**	0.45**
	*Depression*	0.66**	0.64**	0.54**
BDI-II	*Total*	0.67**	0.69**	0.54**
	*Cognitive*	0.64**	0.65**	0.52**
	*Affective*	0.67**	0.68**	0.54**
	*Somatic*	0.60**	0.62**	0.47**
CDI	*Total*	0.73**	0.67**	0.62**
	*Negative Mood*	0.42**	0.39**	0.35**
	*Interpersonal Difficulties*	0.63**	0.63**	0.49**
	*Ineffectiveness*	0.65**	0.57**	0.57**
	*Anhedonia*	0.68**	0.54**	0.64**
	*Negative Self-Esteem*	0.42**	0.36**	0.37**
DERS	*Total*	0.63**	0.59**	0.52**
	*Strategies*	0.55**	0.52**	0.46**
	*Non acceptance*	0.38**	0.40**	0.28**
	*Impulse*	0.45**	0.43**	0.37**
	*Goals*	0.38**	0.37**	0.31**
	*Awareness*	0.35**	0.24**	0.36**
	*Clarity*	0.54**	0.53**	0.43**

### Discriminant validity

The discriminant validity was established by elaborating on the associations between INQ-15 with BRFL which has an inverse concept, while BRFL was used to assess reasons for suicide ideations.

Table 3 shows a significant negative correlation between the total INQ-15 and BRFL scores. This implies that participants with higher scores on INQ-15 tend to score lower in BRFL and vice versa.

**Table 3:** Correlation coefficients between INQ with BRFL and its subscales.

	BRFL	BRFL	BRFL	BRFL	BRFL	BFRL
	total	FSD	MO	SCB	RF	FS
INQ_total	-0.34**	0.16**	-0.28**	-0.52**	-0.49**	-0.03
INQ_PB	-0.33**	0.17**	-0.29**	-0.52**	-0.47**	-0.03
INQ_TB	-0.28**	0.11**	-0.22**	-0.42**	-0.40**	-0.01

Note: FSD = Fear of Social Disapproval; MO = Moral Objections; SCB = Survival and Social Beliefs; RF = Responsibility to Family; FS = Fear of Suicide

** p < 0.01

### Factorial validity

**Fig 1:**
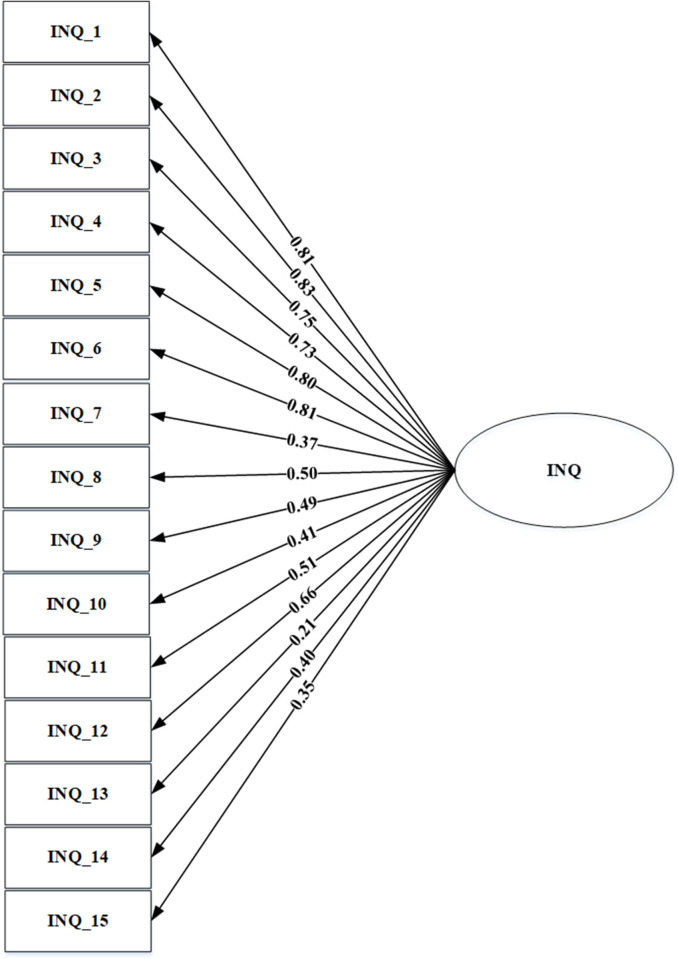
Single factor model.

**Fig 2:**
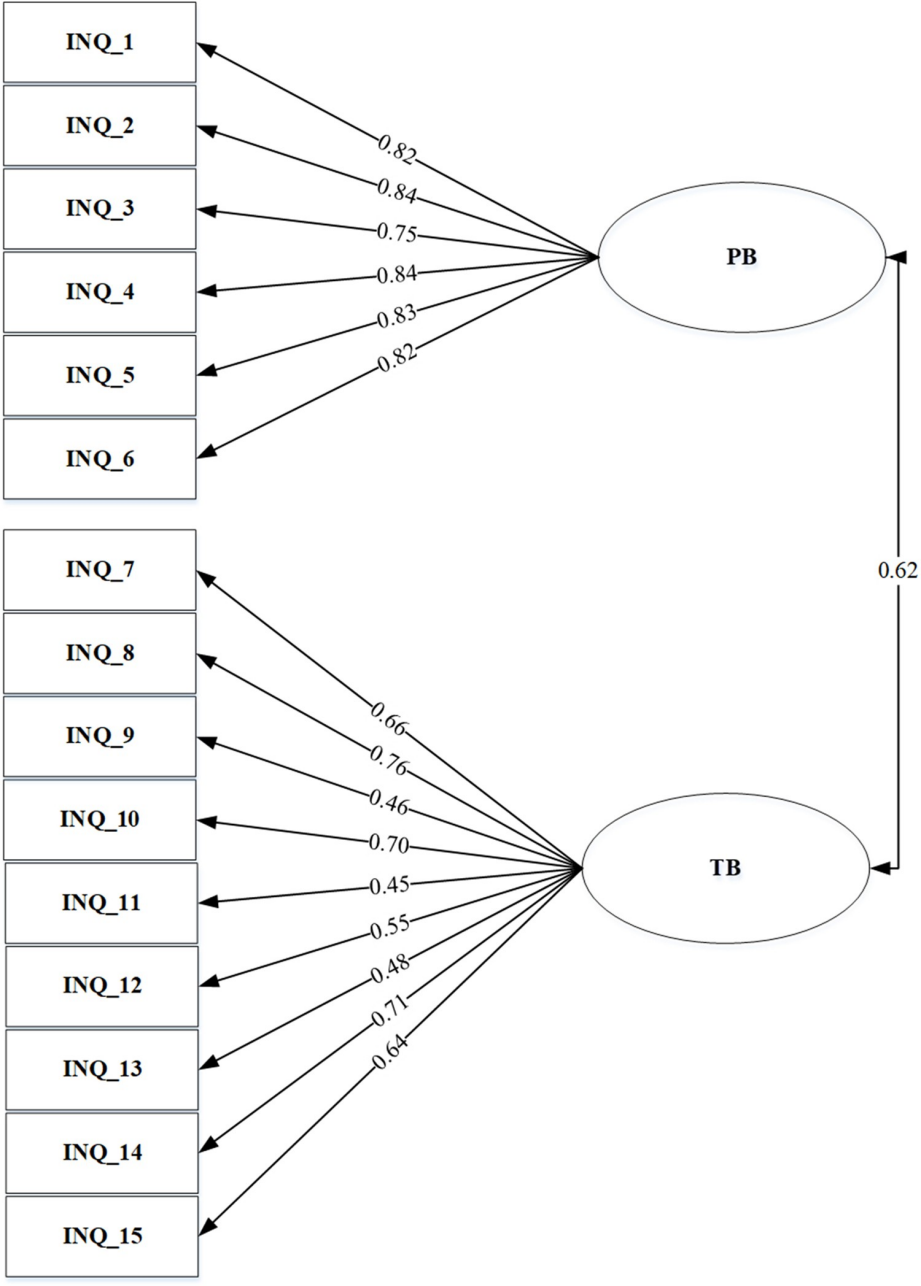
Two factors model.

We tested two-factor models of INQ using confirmatory factors. Model 1 was a single-factor model, and model 2 was two factors model consisting of two subscales, PB and TB. All loading factors of the items were significant for model 1 and model 2. Figs 1 and 2 shows that the values of factor loadings of model 2 were higher (0.45–0.84) than model 1. The result of the two models was a reasonably good fit for the data. The goodness of fit indices of the factor model is explained in Table 4.

**Table 4:** The goodness of fit indices of the factor model.

Model factor	($\frac{\chi^{2}}{df}$)	AGFI	NNFI	CFI	RMSEA	SRMR
Model 1	1.96	0.96	0.99	0.99	0.03	0.04
Model 2	5.57	0.90	0.93	0.95	0.07	0.08

## Discussion

This research examined the psychometric properties of the INQ-15 in the Indonesian language among college and high school students. The results indicate that INQ-15 has good to excellent internal consistency and test-retest reliability. Regarding the validity indicators, it also showed satisfactory content and concurrent validity, as well as further supported the one-factor model for its factorial validity. This result is similar to previous research in Asian countries such as Singapore with Cronbach’s Alpha = 0.93 [21].

The two factors model of the INQ-15 was confirmed to be adequate based on the CFA value. The results are similar to previous research conducted in Asian countries such as Singapore and Korea [21, 25]. The RMSEA value was less than 0.08 and considered adequate, while CFI values were more than 0.90. Furthermore, the average loading factors for PB were higher than TB. Several previous investigations also found similar results, suggesting that PB has a more significant impact on suicidal ideation than TB, especially in the Asian population of young adults and college students [21, 51]. The correlation between PB and TB was moderate (0.62), which is also similar to previous reports by Teo with 0.66 and Van Orden 0.67 [10, 21].

The results also show that the INQ-15 correlated with the construct of psychological distress r = 0.52, depression in young adults r = 0.67, and adolescents r = 0.73, as well as difficulties in emotional regulation r = 0.63. Previous research in Indonesia in 2019 found that interpersonal needs and depressive symptoms have a significant effect on suicidal ideation [52]. Moreover, Spinola and Campos highlighted the importance of assessing interpersonal needs, in relation to depression, with suicidal ideation in young adults [53]. The domain of PB had a stronger correlation than TB with the construct of psychological distress, depression, and difficulties in emotional regulation. This result is concurrent with Wong, et al who conducted a research on the Asian population [51], as well as other investigations which found that the domain of PB had a significant correlation with depression. This indicates that perceived burdensomeness can be a predisposing risk factor to depression and suicidal ideation, especially within a family environment [10, 21, 51]. There was also a significant negative correlation between total INQ-15 and total BRFL score, indicating that reasons to live such as moral values, responsibility to family, survival, and coping beliefs might reduce the risk of suicidal ideation in adolescents and young adults. Moscardini, et al stated that reasons for living acted as a protective factor for suicidal ideation, while thwarted interpersonal needs can increase suicidal ideation [54]. Therefore, reasons for living such as moral education, coping strategies, and family support might have a significant impact on preventing suicide.

## Conclusion and limitations

The main limitation of this research is that the data were taken during the COVID-19 pandemic period. Therefore, this situation might have affected the interpersonal needs of the participants, especially adolescents and young adults. Further investigations into the impact of the COVID-19 pandemic on the interpersonal needs of young adults and adolescents are needed. The other limitations are that it focused on young adults and adolescents only, hence, the results cannot be generalized across age groups, or to clinical populations in Indonesia. Future studies are recommended to replicate the current findings in more heterogeneous samples across Asia. Nevertheless, the Indonesian version of the INQ-15 was proven to be valid and reliable in adolescents and young adults population. Therefore, policy recommendations for this screening can be implemented in educational institutions as an effort to prevent suicide programs. Additionally, the results contribute potentially to the development of suicide screening and prevention programs in Indonesia or other Asian countries.

## Supporting information

S1 File Permission to use and validate INQ in Indonesian language. (PDF)

S2 File Indonesian version of INQ. (DOCX)
