# Supplementary material for: Psychometric evaluation of the interpersonal needs questionnaire in the Indonesian language
Source: PLoS One. 2022 Dec 16;17(12):e0279272. doi: 10.1371/journal.pone.0279272 (PMC9757555; doi:10.1371/journal.pone.0279272)
Supplement: S2 File — (DOCX) [file pone.0279272.s002.docx]

**Interpersonal Needs Questionnaire (INQ)**

Pertanyaan-pertanyaan berikut meminta Anda untuk memikirkan diri sendiri dan orang lain. Harap jawab setiap pertanyaan dengan menggunakan keyakinan dan pengalaman Anda saat ini, BUKAN apa yang Anda anggap benar secara umum, atau apa yang mungkin benar bagi orang lain. Harap dasarkan respons Anda pada bagaimana perasaan Anda baru-baru ini. Gunakan skala peringkat untuk menemukan nomor yang paling cocok dengan perasaan Anda dan lingkari nomor itu. Tidak ada jawaban benar atau salah: kami tertarik pada apa yang Anda pikirkan dan rasakan.

1 : Sama sekali tidak menggambarkan diri saya

2 : Tidak menggambarkan diri saya

3 : Agak tidak menggambarkan diri saya

4 : Agak menggambarkan diri saya

5 : Cukup menggambarkan diri saya

6 : Menggambarkan diri saya

7 : Sangat menggambarkan diri saya

1. Akhir-akhir ini, orang-orang dalam hidup saya akan lebih baik jika saya tidak ada
2. Akhir-akhir ini, orang-orang dalam hidup saya akan lebih bahagia tanpa saya
3. Akhir-akhir ini, saya berpikir saya adalah beban bagi masyarakat
4. Akhir-akhir ini, saya berpikir kematian saya akan membuat lega orang-orang dalam hidup saya
5. Akhir-akhir ini, saya berpikir orang-orang dalam hidup saya berharap mereka dapat terbebas dari saya
6. Akhir-akhir ini, saya berpikir saya memperburuk keadaan orang-orang dalam hidup saya
7. Akhir-akhir ini, orang-orang peduli kepada saya
8. Akhir-akhir ini, saya merasa diterima oleh orang-orang di sekitar saya
9. Akhir-akhir ini, saya jarang berinteraksi dengan orang-orang yang peduli kepada saya
10. Akhir-akhir ini, saya beruntung memiliki banyak teman yang peduli dan mendukung saya
11. Akhir-akhir ini, saya merasa terputus hubungan dengan orang lain
12. Akhir-akhir ini, saya sering merasa seperti orang asing ketika berkumpul dengan orang lain
13. Akhir-akhir ini, saya merasa bahwa ada orang-orang tempat saya mengadu ketika saya membutuhkannya
14. Akhir-akhir ini, saya merasa dekat dengan orang lain
15. Akhir-akhir ini, saya memiliki setidaknya satu interaksi yang memuaskan setiap harinya
