# Supplementary material for: Psychometric evaluation of the interpersonal needs questionnaire in the Indonesian language
Source: PLoS One. 2022 Dec 16;17(12):e0279272. doi: 10.1371/journal.pone.0279272 (PMC9757555; doi:10.1371/journal.pone.0279272)
Supplement: S1 File — (PDF) [file pone.0279272.s001.pdf]

Fredrick Purba <fredrick.purba@unpad.ac.id>

Re: Permission to use and validate the INQ in Indonesia

1 message

Van Orden, Kimberly <Kimberly_Vanorden@urmc.rochester.edu> Sat, Feb 22, 2020 at 3:00 AM
To: Fredrick Purba <fredrick.purba@unpad.ac.id>, Efi Fitriana <efi.fitriana@unpad.ac.id>
Cc: "janakiraman@psy.fsu.edu" <janakiraman@psy.fsu.edu>, "Silva, Caroline" <Caroline_Silva@urmc.rochester.edu>, Thomas Joiner <joiner@psy.fsu.edu>

Hello—you are welcome to use the measure, there is no cost. I would suggest the 15 item version.

I cc'ed my colleagues to see if they are aware of any relevant translations.

Best,

Kim

--

Kim Van Orden, Ph.D.

Associate Professor

Department of Psychiatry
University of Rochester School of Medicine and Dentistry
300 Crittenden Blvd, Box Psych Research, Rochester, NY 14642
P: 585-275-5176; F: 585-276-2065; Office # 4.9266
Kimberly_vanorden@urmc.rochester.edu

Helping Older People Engage (HOPE) Lab

Suicide Prevention Resource Center Steering Committee Member

From: Fredrick Purba <fredrick.purba@unpad.ac.id>
Date: Friday, February 21, 2020 at 1:30 AM
To: Kim Van Orden <Kimberly_Vanorden@URMC.Rochester.edu>
Cc: Efi Fitriana <efi.fitriana@unpad.ac.id>
Subject: Permission to use and validate the INQ in Indonesia

Dear Prof Van Orden,

I am involved in a research team that develops an intervention program of suicidal thoughts prevention for adolescents and adults in Indonesia. We plan to use the Interpersonal Theory of Suicide as the theory and therefore to use the Interpersonal Needs Questionnaire as part of the measurement tools. We check and nothing found on their Indonesian version. I have several questions:

1. There are 5 versions (10-, 12-, 15-, 18-, and 25-items). Some literature suggests that the 15-items version is the best to use. Do you have any suggestions about which one to use?
2. Would it be possible to adapt the questionnaires into the Indonesian language? What will be the procedure to do so?
3. Furthermore, would it be possible to use it for free in Indonesia? Thanks in advance!

Looking forward to hearing from you!

Salam / regards,

Fredrick Dermawan Purba, PhD

Department of Developmental Psychology

Universitas Padjadjaran

Jatinangor, Indonesia
